# The evolution of body composition in oncology—epidemiology, clinical trials, and the future of patient care: facts and numbers

**DOI:** 10.1002/jcsm.12379

**Published:** 2019-01-13

**Authors:** Justin C. Brown, Elizabeth M. Cespedes Feliciano, Bette J. Caan

**Affiliations:** ^1^ Pennington Biomedical Research Center Baton Rouge LA USA; ^2^ Stanley S. Scott Cancer Center Louisiana State University Health Sciences Center New Orleans LA USA; ^3^ Kaiser Permanente Northern California Oakland CA USA

**Keywords:** Obesity paradox, Computed tomography, Cancer, Randomized trial, Cohort study, Metabolism

## Abstract

There is growing interest from the oncology community to understand how body composition measures can be used to improve the delivery of clinical care for the 18.1 million individuals diagnosed with cancer annually. Methods that distinguish muscle from subcutaneous and visceral adipose tissue, such as computed tomography (CT), may offer new insights of important risk factors and improved prognostication of outcomes over alternative measures such as body mass index. In a meta‐analysis of 38 studies, low muscle area assessed from clinically acquired CT was observed in 27.7% of patients with cancer and associated with poorer overall survival [hazard ratio: 1.44, 95% CI: 1.32–1.56]. Therapeutic interventions such as lifestyle and pharmacotherapy that modify all aspects of body composition and reduce the incidence of poor clinical outcomes are needed in patients with cancer. In a meta‐analysis of six randomized trials, resistance training exercise increased lean body mass assessed from dual‐energy X‐ray absorptiometry [mean difference (MD): +1.07 kg, 95% CI: 0.76–1.37; *P* < 0.001] and walking distance [MD: +143 m, 95% CI: 70–216; *P* < 0.001] compared with usual care control in patients with non‐metastatic cancer. In a meta‐analysis of five randomized trials, anamorelin (a ghrelin agonist) significantly increased lean body mass [MD: +1.10 kg, 95% CI: 0.35–1.85; *P* = 0.004] but did not improve handgrip strength [MD: 0.52 kg, 95% CI: −0.09–1.13; *P* = 0.09] or overall survival compared with placebo [HR: 0.99, 95% CI: 0.85–1.14; *P* = 0.84] in patients with advanced or metastatic cancer. Early screening to identify individuals with occult muscle loss, combined with multimodal interventions that include lifestyle therapy with resistance exercise training and dietary supplementation combined with pharmacotherapy, may be necessary to provide a sufficient stimulus to prevent or slow the cascade of tissue wasting. Rapid, cost‐efficient, and feasible methods to quantify muscle and adipose tissue distribution are needed if body composition assessment is to be integrated into large‐scale clinical workflows. Fully automated analysis of body composition from clinically acquired imaging is one example. The study of body composition is one of the most provocative areas in oncology that offers tremendous promise to help patients with cancer live longer and healthier lives.

## Introduction

There is growing interest from the oncology community to understand how body composition measures can be used to improve cancer treatment and survivorship care for the 18.1 million individuals diagnosed with cancer annually.^1^ Specifically, there is an emergent recognition that body mass index (BMI, weight in kilograms divided by the square of height in meters) is not adequate to identify patients who are at risk for adverse health outcomes due to poor muscle health or excess adiposity, nor does BMI accurately classify the distribution of adiposity.^2^ Historically, oncology has appreciated the deleterious prognostic effect of involuntary weight loss.^3^ Recent observational studies demonstrate that muscle and adipose tissue distribution are risk factors for clinical outcomes such as post‐operative complications, chemotherapy‐related toxicity, and overall survival in patients with cancer.^4^, ^5^ Underscoring the critical need for the evaluation of body composition in oncology, patients with cancer are often older adults who have experienced age‐related alterations in body composition that may be further exacerbated by cancer and cancer treatments.^6^, ^7^ The field is eager to identify therapeutic interventions that modify body composition and reduce the incidence of poor clinical outcomes in this population.^8^ Furthermore, a pre‐requisite to using body composition measures in oncology practice is to seamlessly integrate their assessment into the clinical oncology workflow.^9^ The purpose of this paper is to provide a concise overview of the *facts and numbers* that relate to the (1) epidemiology that describes the relationship between body composition and cancer prognosis, (2) evidence from clinical trials with body composition endpoints in patients with cancer, and (3) evidence describing how body composition can be integrated into oncology practice to guide patient care.

## The epidemiology of body composition in cancer

### Body mass index

Body mass index is often used as a proxy measure of total adiposity. Among adults, overweight is defined as a BMI of 25.0–29.9 kg/m^2^ and obesity as a BMI of ≥30 kg/m^2^. Worldwide, 1.9 billion adults are overweight; of these, 650 million are obese.^10^ It is estimated that 1 in 11 (9%) incident cancers diagnosed in North America and Europe is attributable to obesity.^11^ The International Agency for Cancer Research reviewed ≥1000 observational studies and concluded that there is convincing evidence that a high BMI is associated with an increased risk of developing 13 types of cancer.^12^

**Figure 1:**
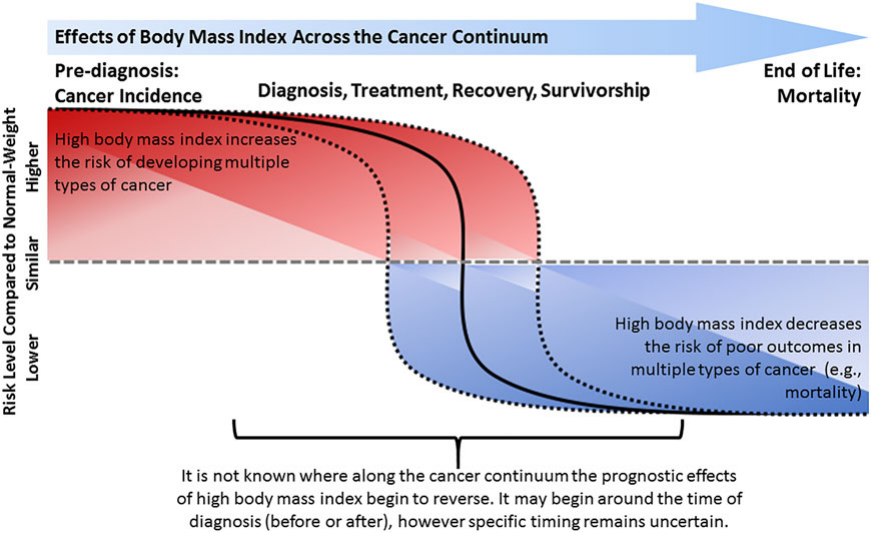
The effects of body mass index across the cancer continuum. Higher body mass index before diagnosis increases the risk of developing multiple cancers and higher body mass index at or after diagnosis lowers the risk of dying from multiple cancers. The interval along the cancer continuum where the higher body mass index begins to switch from a deleterious risk factor to an advantageous risk factor, and why, is not yet known.

In contrast to cancer incidence, the association between BMI and cancer prognosis (e.g. cancer‐specific survival or overall survival) is less consistent, and for many malignancies, overweight or obesity is associated with a survival advantage (Figure 1). The association between BMI at diagnosis and prognosis may depend on cancer type, stage at diagnosis, age, sex, and type of treatments utilized. For example, in a pooled analysis of 22 randomized therapeutic treatment trials that included 11 724 patients with cancer, 67% were overweight/obese (BMI ≥25 kg/m^2^) at the time of enrolment (e.g. cancer diagnosis), and this was independently associated with improved overall survival in patients with several types of malignancies, including bladder [HR: 0.69; *P* = 0.02], gastrointestinal stromal tumours [HR: 0.73; *P* = 0.006], non‐small cell lung cancer [HR: 0.76; *P* = 0.01], and prostate cancer [HR: 0.79; *P* = 0.01].^13^ Other studies among a variety of cancer types have reported that a higher BMI is associated with improved overall survival (Table 1). The observed survival benefit associated with a higher BMI has historically been referred to as the obesity paradox^14^; however, it has been advised that this label be abandoned, given its inadequacy as a scientific descriptor.^15^

**Table 1:** Association between body mass index (BMI) at cancer diagnosis and overall survival

Cancer site or type	Relative risk of the overweight/obese BMI category evaluated vs. normal BMI (95% CI)
Myeloid leukemia^94^	0.47 (0.26–0.82)
Non‐metastatic colorectal^65^	0.52 (0.35–0.77)
Metastatic melanoma^a^, ^29^	0.72 (0.57–0.91)
Lymphoma^95^	0.76 (0.67–0.86)
Gastric^96^	0.76 (0.59–0.99)
Renal^97^	0.84 (0.73–0.95)
Metastatic colorectal^28^	≈0.90 (≈0.85–0.95)

a Among patients treated with targeted therapy

There are several proposed explanations for the observation that higher BMI is associated with improved overall and cancer‐specific survival in patients with cancer.^16^, ^17^, ^18^ Some of these explanations relate to methodological concerns of study design and statistical analysis such as selection biases, unmeasured or residual confounding, and illness‐related weight loss. However, when these methodological concerns are empirically tested, many are not substantiated and the previously observed associations persist.^17^, ^18^, ^19^, ^20^, ^21^, ^22^ Other explanations involve BMI being too crude a measure to be useful at the individual patient level.^23^ BMI does not differentiate lean mass from adipose mass, nor does it describe regional adipose tissue deposition (e.g. visceral vs subcutaneous).^24^ Compared with bioelectrical impedance analysis and dual‐energy X‐ray absorptiometry to diagnose excess adiposity, BMI has poor sensitivity (36–49%) which results in high misclassification rates,^25^ and this limitation is worsened in older adults, particularly among males (32–38% sensitivity).^26^ This observation may explain, in part, why many studies have observed a statistical interaction between BMI and sex, such that a high BMI is associated with improved overall survival among male but not female patients with cancer. Such an interaction has been observed in non‐metastatic colon cancer (*P*_interaction_ = 0.012),^27^ metastatic colorectal cancer (*P*_interaction_ < 0.001),^28^ and metastatic melanoma (*P*_interaction_ = 0.01).^29^ Moreover, in a pooled analysis of solid and hematologic malignancies, sex‐stratified analysis demonstrated that a BMI ≥25 kg/m^2^ was associated with improved overall survival in males [HR: 0.82; *P* = 0.003] but not in females [HR: 1.04; *P* = 0.86].^13^ The totality of these data suggests that BMI alone is insufficient and that more accurate measures of muscle and adipose tissue distribution may improve prognostication of outcomes.^30^, ^31^

### Body composition

At the time of diagnosis, patients with cancer often complete radiologic measures such as computed tomography or magnetic resonance imaging to characterize the primary tumour and identify distant metastatic foci. Computed tomography and magnetic resonance imaging are gold‐standard techniques to quantify body composition,^32^ and the strengths and weaknesses of these modalities in oncology have been reviewed.^33^ A single abdominal cross‐sectional computed tomography image at the third lumbar vertebra provides an accurate estimate of whole‐body skeletal muscle (*R* ^2^ = 0.855; *P* < 0.001) and adipose tissue (*R* ^2^ = 0.927; *P* < 0.001) volumes.^34^ Existing clinically acquired images from patients with cancer have provided a rich source of data for investigators to quantify measures of body composition and their prognostic importance in patients with cancer.

Low abdominal muscle cross‐sectional area measured using computed tomography at the time of diagnosis in patients with cancer is associated with poor prognosis. In a meta‐analysis of 38 studies that included 7843 patients with solid tumours, low muscle cross‐sectional area was observed in 27.7% of patients with cancer and associated with poorer overall survival [HR: 1.44, 95% CI: 1.32–1.56, *P* < 0.001], cancer‐specific survival [HR: 1.93, 95% CI: 1.38–2.70, *P* < 0.001], and disease‐free survival [HR: 1.16, 95% CI: 1.00–1.30, *P* = 0.014].^5^ The deleterious effects of low muscle area on overall survival were similar between non‐metastatic [HR: 1.54, 95% CI: 1.31–1.79, *P* < 0.001] and metastatic disease [HR: 1.37, 95% CI: 1.21–1.56, *P* < 0.001], and consistent across various tumour types. This observation has been confirmed in two large cohort studies of 3241 females with breast cancer and 3262 males and females with colorectal cancer, where low abdominal muscle cross‐sectional area was observed in 34–42% of patients, and this was independently associated with a 27–41% higher risk of overall mortality.^35^, ^36^ Multiple meta‐analyses have now summarized the prognostic importance of low muscle area in a variety of cancer sites, such as colorectal [HR: 1.63, 95% CI: 1.24–2.14; *P* < 0.01],^37^ gastric [HR: 1.70, 95% CI: 1.45–1.99; *P* < 0.01],^38^ esophageal [HR: 1.70, 95% CI: 1.33–2.17; *P* < 0.001],^39^ and hepatocellular carcinoma [HR: 1.95, 95% CI: 1.60–2.37; *P* < 0.001].^40^ In addition to low muscle area, low muscle radiodensity (indicative of intramyocellular lipid)^41^ is associated with poorer overall survival in patients with colorectal cancer [HR: 1.61, 95% CI: 1.36–1.90],^42^ non‐small cell lung cancer [HR: 1.19, 95% CI: 1.07–1.33],^43^ B‐cell lymphoma [HR: 2.52, 1.40–4.54],^44^ and endometrial cancer [HR: 2.03, 95% CI: 1.09–3.78].^45^ Muscle cross‐sectional area and radiodensity may each be independent prognostic factors for overall and cancer‐specific mortality. For example, among 1924 patients with stage I–III colorectal cancer, the deterioration of muscle area [HR: 2.15, 95% CI: 1.59–2.92; *P* < 0.001] and muscle radiodensity [HR: 1.61, 95% CI: 1.20–2.15; *P* = 0.002] were independently prognostic of all‐cause and cancer‐specific mortality.^46^ There are also emerging data that excess visceral adiposity may be associated with overall survival; however, these data are mixed, with higher visceral adiposity associated with poorer overall survival in colorectal and pancreatic cancer, but improved overall survival in renal cell carcinoma.^47^

The use of body composition quantified with clinical imaging is, however, not without limitation. One of the principal methodological challenges is disentangling the physiological, prognostic, and statistical interactions between muscle and adiposity. Patients with higher BMI have more muscle mass on the absolute (kg) scale, but less muscle mass on the relative (%) scale, compared with those with a lower BMI.^48^ It is not yet established if absolute muscle mass or the relative proportion of muscle mass compared with total adiposity is a superior predictor of outcome in patients with cancer. To address this issue, some studies have statistically adjusted for the complementary body composition tissue. For example, low muscle cross‐sectional area was associated with poorer overall survival in patients with breast cancer [HR: 1.30, 95% CI: 1.10–1.54, *P* < 0.001], and after adjustment for total adiposity, the magnitude of the association was strengthened [HR: 1.41, 95% CI: 1.18–1.69].^35^ Another approach is to model the joint effects of muscle and adiposity using phenotype methods. For example, the combined presence of both low muscle cross‐sectional area and high total adiposity was associated with poorer overall survival in patients with colorectal cancer [HR: 1.40, 95% CI: 1.03–1.90] compared with those with adequate muscle and low adiposity.^36^ There is emerging interest in the co‐occurrence of low muscle and excess adiposity, known as sarcopenic obesity,^49^ and additionally osteoporosis known as osteo‐sarcopenic obesity.^50^ Many studies to date have not accounted for the potential opposing or joint prognostic effects that may exist between muscle and adipose tissue.

### The need for randomized clinical trials to modify body composition in cancer

A robust pipeline of randomized clinical trials will advance this research area.^8^, ^51^ It is unknown if body composition is causally related to the occurrence of clinical events, such as post‐operative complications, chemotherapy‐related toxicities, disease recurrence or progression, and overall survival in patients with cancer. Randomized clinical trials are necessary to determine if the effects of body composition on clinical endpoints are both causal, and, more importantly, reversible through intervention. The physiological mechanisms that link body composition with clinical events are multi‐factorial and include metabolic alterations such as inflammation, oxidative stress, myostatin activation, and insulin resistance which promote a catabolic state, and are worsened with physical inactivity and nutritional deficiency.^52^, ^53^ Herein, we describe lifestyle and pharmacotherapies that have been examined in patients with cancer for the purposes of manipulating body mass or body composition and proposed recommendations for the next generation of randomized clinical trials.

### Lifestyle therapy for body composition management

Lifestyle therapy, including exercise and dietary modification, are efficacious interventions to influence body composition in patients with cancer. The primary modality to quantify body composition in randomized controlled trials has been dual‐energy X‐ray absorptiometry. In a meta‐analysis of six randomized controlled trials that included patients with early stage breast and prostate cancer, progressive resistance training exercise increased lean body mass [mean difference (MD): +1.07 kg, 95% CI: 0.76–1.37, *P* < 0.001] and decreased body fat [MD: −2.08%, 95% CI: −3.46 to −0.70, *P* = 0.003] compared with usual care control during an average of 18 weeks.^54^ Resistance training exercise also improved functional outcomes including muscle strength of the lower [MD: +14.6 kg, 6.3–22.8, *P* < 0.001] and upper [MD: +6.9, 95% CI: 4.8–9.0, *P* < 0.001] extremities, and walking distance [MD: +143 m, 95% CI: 70–216, *P* < 0.001]. Similar magnitude of benefit has been summarized in meta‐analyses for patients with breast and prostate cancer.^55^, ^56^ In a meta‐analysis of 22 randomized control trials among healthy adults, the combination of resistance exercise plus protein supplementation increased lean body mass vs. resistance exercise plus placebo [MD: +0.69 kg, 95% CI: 0.47–0.91, *P* < 0.001].^57^ An ongoing randomized trial is examining the efficacy of resistance exercise and protein supplementation to improve lean mass and reduce chemotherapy‐related dose‐limiting toxicities in patients with colon cancer (ClinicalTrials.gov Identifier: NCT03291951). Aerobic exercise significantly reduces visceral adiposity in patients with colon cancer [MD: −2.7 cm^2^ per 60 min of aerobic exercise; *P* < 0.001] while preserving lean mass over 6 months.^58^ Additional benefits of exercise for patients with cancer have been described.^59^, ^60^ There are limited data testing the effects of specific dietary interventions on body composition in patients with cancer; many studies have focused on weight loss using caloric restriction.^61^, ^62^ Randomized controlled trials have demonstrated that caloric restriction reduces body mass [MD: −5.8%, 95% CI: −3.8 to −7.8; *P* < 0.001] and fat mass [MD: −3.2 ± 0.7 kg, *P* < 0.001] in patients with cancer; however, these changes are also accompanied by declines in lean body mass [MD: −1.7 ± 0.4 kg, *P* < 0.001].^63^ A randomized phase III trial is evaluating the effects of purposeful weight loss on distant disease‐free survival among 3136 overweight and obese women with breast cancer.^64^ Until definitive evidence emerges, the benefits of weight loss for overweight and obese patients with cancer remain contested.^65^

### Pharmacotherapy for body composition management

Pharmacotherapy may offer benefit for the management of body composition in patients with advanced or metastatic cancer.^66^ Therapies that utilize or target ghrelin,^67^, ^68^ androgen receptors,^69^ interleukin‐1α,^70^, ^71^ β receptor blockade,^72^ testosterone,^73^ and myostatin^74^ have been evaluated in randomized clinical trials. Among therapies with phase III data, none have received regulatory approval for clinical use. Anamorelin, a ghrelin receptor agonist, has extensive clinical data from randomized controlled trials evaluating therapeutic efficacy in patients with inoperable stage III or IV non‐small‐cell lung cancer and cachexia (defined as ≥5% weight loss within 6 months or BMI <20 kg/m^2^).^68^ In a meta‐analysis of five randomized controlled trials, anamorelin significantly increased lean muscle mass compared with placebo [MD: +1.10 kg, 95% CI: 0.35–1.85; *P* = 0.004]. However, handgrip strength was not improved [MD: 0.52 kg, 95% CI: −0.09 − 1.13; *P* = 0.09], and overall survival did not differ between randomized groups [HR: 0.99, 95% CI: 0.85–1.14; *P* = 0.84].^75^ The most common treatment‐related adverse events were hyperglycemia and gastrointestinal disorders. Despite the success of these therapies to increase lean body mass, their inability to influence functional measures has led to failure of regulatory approval. Many studies to date have investigated pharmacotherapy in patients with advanced or metastatic cancer with established cachexia. The risk to benefit ratio of pharmacotherapy, including weight loss agents in patients with early stage cancer who have not yet developed cachexia, has not been comprehensively evaluated.

### The future of randomized clinical trials

**Figure 2:**
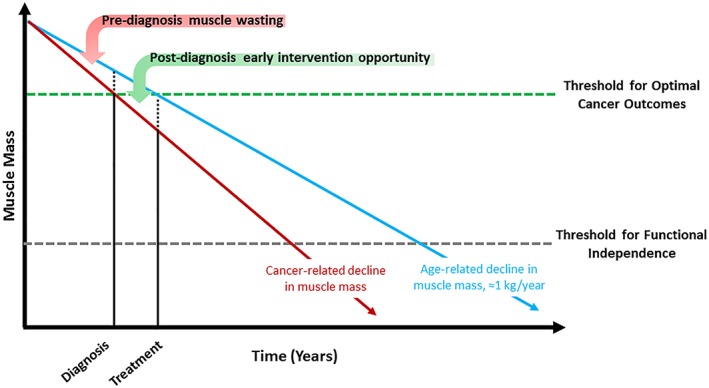
Schematic underscoring the hypothesized importance of early multimodal intervention to preserve muscle mass in patients with cancer. Relative to healthy adults, patients with cancer may experience pre‐diagnosis muscle wasting, and after diagnosis, this muscle wasting may be accelerated from cancer treatments. Early identification and multimodal intervention may help to retard the rate of decline in muscle mass and thereby prevent patients from falling below the critical threshold of muscle mass that is necessary for optimal cancer outcomes.

If body composition has a causal effect on the incidence of clinical events such as overall survival in patients with cancer, multimodal interventions may be necessary to provide a sufficient therapeutic stimulus to impact disease progression. Early screening to identify individuals with occult muscle loss, combined with lifestyle therapy including resistance exercise training and dietary supplementation (e.g. protein), and pharmacotherapy may be necessary to provide a sufficient stimulus to prevent or slow the cascade of tissue wasting (Figure 2).^76^ To date, most trials of lifestyle therapy have focused on patients with early stage breast and prostate cancer, whereas trials of pharmacotherapy have focused on patients with advanced or metastatic lung and gastrointestinal cancer who have overt cachexia. Early intervention to prevent the deterioration of body composition may be more effective than efforts to improve body composition in patients with established cachexia. For example, resistance exercise among patients with early‐stage breast cancer attenuated the rate of decline of appendicular lean mass [−0.01 vs −0.08 kg/m^2^; *P* = 0.041],^77^ and the deterioration of physical functioning (relative risk: 0.49, 95% CI: 0.25–0.96; *P* = 0.04) over 12 months.^78^ Furthermore, lifestyle therapy and pharmacotherapy may have complementary effects, that when used together have the potential to increase lean mass, increase muscle strength, and obviate functional and clinical decline. An example of a multimodal intervention that is being tested within an ongoing phase III trial includes non‐steroidal anti‐inflammatory medication, eicosapentaenoic acid, resistance and aerobic exercise, and dietary counselling with oral nutritional supplements to prevent weight loss and the deterioration of body composition in patients with advanced or metastatic cancer.^79^ Studies to date have focused on the importance of muscle; however, greater attention to the prognostic effects of excess adiposity and the development of interventions with the potential to simultaneously increase muscle and reduce adiposity may be of critical importance. Continued efforts to investigate the efficacy of multimodal interventions are urgently needed to advance this area.

### Bridging the gap to understand how body composition can be integrated into patient care

Despite the absence of randomized clinical trials, opportunities exist to integrate body composition measures into oncology care to guide clinical decision making. Despite interest from radiologists to quantify body composition,^80^ several barriers exist to seamless integration into clinical workflows. Automated and semi‐automated methods to quantify body composition using clinical imaging, such as computed tomography and magnetic resonance imaging, have been developed.^81^, ^82^ Diagnostic imaging is already available clinically on large numbers of patients, but it is not standard of care in all cancers and the validity of these methods has not been extensively studied. Other modalities to quantify body composition that can be implemented within clinical settings are also being explored. Ultrasound is a valid, safe, and portable method to quantify various muscle parameters such as volume, cross‐sectional area, and thickness. Several studies have demonstrated that ultrasound measures of muscle correlate well with lean body mass assessed using dual‐energy X‐ray absorptiometry among older adults (*R* ^2^ = 0.929–0.955).^83^ Non‐imaging methods, such as bioelectrical impedance analysis and creatine (methyl‐d3) dilution, have also been evaluated. Bioelectrical impedance analysis and the related reactance and resistance measures are associated with overall survival in patients with cancer; however, these measures are limited by high inter‐patient variability that is influenced by hydration status.^84^ Creatine dilution, measured by the enrichment of urinary D3‐creatinine 3–5 days after ingestion, is correlated with total muscle mass quantified using magnetic resonance imaging (*r* = 0.868).^85^ Any method used to screen for low muscle or excess adiposity in oncology practice will need to be predictive of important cancer outcomes and easily implemented within existing clinical workflows (e.g. automated or rapid assessments with standardized risk thresholds).

Body composition can also be used as a prognostic biomarker to identify patients who are most likely to experience adverse events and toxicities from cancer‐directed therapy. For example, low muscle mass is associated with an increased risk of major post‐operative surgical complications [HR: 1.40, 95% CI: 1.20–1.64, *P* < 0.001],^86^ and chemotherapy‐related toxicity in patients with non‐metastatic colorectal [odds ratio: 2.34, 95% CI: 1.04–5.24, *P* = 0.03],^87^ and metastatic breast cancer [57 vs 18%, *P* = 0.02].^88^ Chemotherapy dosing currently utilizes body surface area, which does not account for the distribution of lean and adipose tissue throughout the body.^89^ For as long as clinical trials continue to use body surface area, it is likely that chemotherapy dosing in clinical practice will continue to be guided by this measure; however, some studies have begun to explore the use of body composition to guide chemotherapy dosing in the setting of advanced cancer (ClinicalTrials.gov Identifier: NCT01624051).^90^, ^91^ Until clinical trial data emerge, oncologists may use measures of body composition to identify patients who may benefit from preventive interventions (e.g. pegfilgrastim prophylaxis for febrile neutropenia).^92^

## Conclusions

The study of body composition is one of the most provocative areas in oncology. There is growing observational evidence that measures of body composition obtained from clinically acquired imaging are associated with numerous outcomes in patients with cancer. Randomized clinical trials that test multimodal interventions including early identification, lifestyle therapy, and pharmacotherapy may offer the largest potential for clinical benefit. The emergence of automated techniques to quantify body composition will allow for rapid and early intervention of high‐risk patients. Transdisciplinary teams of investigators that span basic, clinical, and population sciences will accelerate the discovery of therapeutics and their translation into clinical practice. The emerging opportunities to integrate body composition measures into oncology offers tremendous promise to help patients with cancer live longer and healthier lives.

## Conflict of interest

None declared.
